# Phenolics from *Chaenomeles speciosa* leaves: Ionic liquid-based ultrasound-assisted extraction, adsorptive purification, UPLC–QqQ–MS/MS quantification, and bioactivity assessment

**DOI:** 10.1016/j.ultsonch.2025.107282

**Published:** 2025-02-16

**Authors:** Mengyang Hou, Chengyuan Lin, Lin Zhu, Zhaoxiang Bian

**Affiliations:** a Centre for Chinese Herbal Medicine Drug Development, Hong Kong Baptist University, Hong Kong 999077, PR China; b School of Chinese Medicine, Hong Kong Baptist University, Hong Kong 999077, PR China

**Keywords:** *Chaenomeles speciosa* leaves, Phenolics, Ionic liquid-based ultrasound-assisted extraction, UPLC–QqQ–MS/MS, Bioactivity

## Abstract

This study aimed to enhance the valorization of *Chaenomeles speciosa* leaves as a sustainable source of bioactive phenolics. An innovative ionic liquid-based ultrasound-assisted extraction (IL-UAE) method was developed for extracting phenolic compounds. Among 10 structurally diverse ILs, [BMIM]Br demonstrated superior extraction performance. Using a combination of single-factor design and response surface methodology (RSM), the optimal parameters for IL-UAE were determined to be the [BMIM]Br concentration of 1.33 mol/L, ultrasonic power of 380 W, extraction time of 10 min, and liquid-to-solid ratio of 22 mL/g. Under these conditions, the yield of *C. speciosa* leaves total phenolics (CSL-TP) was 78.14 ± 0.35 mg/g, which was substantially higher than those obtained via conventional heat reflux and UAE. After extraction, the microstructures of *C. speciosa* leaves were examined using scanning electron microscopy (SEM), which confirmed the effectiveness of IL-UAE. Subsequently, NKA-II resin column chromatography was developed to effectively purify crude CSL-TP extracts, guided by leakage and elution curve evaluations, yielding phenolic extracts with a purity of 75.40 % ± 1.93 %. A UPLC–QqQ–MS/MS method was developed for the quantitative analysis of nine major phenolics in purified CSL-TP extracts. Furthermore, bioactivity assessments demonstrated that the purified CSL-TP extracts efficiently scavenged radicals and effectively inhibited the proliferation of HCT-116 and HT-29 cell lines. These results highlight the potential of *C. speciosa* leaves as a valuable resource for the pharmaceutical and food industries, paving the way for the development of innovative therapeutic products and functional foods.

## 1 Introduction

*Chaenomeles speciosa*, a temperate, deciduous, broad-leaved shrub species belonging to the Rosaceae family, is widely cultivated in East Asia, including China, Korea, and Japan [1]. The fruits of *C. speciosa*, commonly known as Chaenomelis Fructus, are valued for their exceptional nutritional values and potential health benefits. The fruits are a popular choice for producing various delectable and health-promoting food and beverage products, such as preserves, jams, and juices [2]. In traditional Chinese medicine, Chaenomelis Fructus is used as a remedy for rheumatism arthralgia, indigestion, vomiting, and diarrhoea [3]. By contrast, the leaves of *C. speciosa* have received limited attention and are often discarded as agricultural waste despite their potential as a valuable resource with diverse applications. Chemical analyses have revealed that *C. speciosa* leaves contain various bioactive compounds, including phenolics, triterpenes, and amino acids. To date, 39 phenolic compounds, including phenolic acids, flavonols, flavanones, flavones, procyanidins, and ellagitannins, have been identified in *C. speciosa* leaves [4]. Given the growing need for the comprehensive utilisation of *C. speciosa* resources, further exploration of the leaves is essential, with a particular focus on phenolics due to their diverse biological activities.

Conventional methods for extracting natural phenolic compounds, such as heat reflux and decoction, often require prolonged extraction times and can lead to the thermal degradation of target compounds. These drawbacks typically result in low extraction yields and inefficient processes [5], [6]. Although classical solvent extraction is cost-effective and requires simple equipment, commonly used solvents such as water, methanol and ethanol exhibit poor selectivity because of their wide-range solubility. This lack of selectivity limits the extraction efficiency because impurities frequently interfere with the process [7]. In recent years, ionic liquids (ILs) have gained attention as sustainable and effective solvents [8], [9]. Compared with conventional solvents, ILs offer several advantages, including low vapour pressure, strong solubility, and exceptional thermal stability [10]. Notably, ILs, which are often referred to as designer solvents, are highly customisable. Their polarity, solubility, hydrophobicity, or hydrophilicity can be modulated by strategically combining anions and cations or by modifying functional groups, to adjust their physicochemical properties [11]. These unique characteristics make ILs attractive alternatives to conventional solvents, as because they can be tailored to improve the selectivity and efficiency of extraction.

Ultrasound-assisted extraction (UAE) is a leading extraction technique because of its unique mechanism and exceptional performance. UAE improves extraction yields and requires shorter processing times, lower temperatures, less energy, and less amount of solvents [12], [13]. Ultrasound waves, typically generated by ultrasonic probes or bath devices operating at frequencies above 20 kHz, generate physical forces via acoustic cavitation. These forces disrupt plant tissues, enhance mass transfer, and facilitate the release of components into solvents [14]. The proposed technique integrates the advantages of both the UAE and ILs, offering a promising pathway for practical applications [15]. The IL-UAE method, recognised as a novel and synergistic approach, effectively enhances the extraction of valuable phenolic compounds from plants, including *Ficus carica* L. [16], *Curcuma longa* L. [17] and *Apocynum venetum* L. leaves [18].

Advancements in LC–MS technology in recent decades have significantly enhanced the study of natural products by enabling efficient, fast and accurate analysis [19]. UPLC is more advantageous than conventional liquid chromatography because it employs smaller particle size and higher pressure, resulting in faster separation and improved resolution [20]. For targeted analysis, triple quadrupole (QqQ) mass spectrometers are the primary tool for quantification because of their adaptability, which enables easy transitions between various operational modes, making them highly versatile for different analytical applications [21]. The multiple reaction monitoring (MRM) mode is particularly favourable for the quantification of compounds. Using specific transitions from precursor to product ions, MRM minimises interference from endogenous molecules with similar molecular weights [22]. Consequently, the operation of UPLC–QqQ–MS/MS in MRM mode is preferred for the quantification of phenolic compounds [3], [23], [24].

To valorize *C. speciosa* leaves as a source of bioactive phenolic compounds, a green IL-UAE technique was used in this study. The IL-UAE parameters were first optimized to maximize the yield of *C. speciosa* leaves total phenolics (CSL-TP). Subsequently, a UPLC–QqQ–MS/MS method was developed for quantifying major phenolic compounds. Finally, the antioxidant and antiproliferative activities of CSL-TP were evaluated. This study contributes to the comprehensive utilization of *C. speciosa* resources and supports the advancement of sustainable circular economy principles.

## 2 Materials and methods

### 2.1 Plant material

In August 2023, fresh *C. speciosa* leaves were collected from Pingdingshan City, Henan Province. The leaves were air-dried to a constant weight, ground, passed through a 40-mesh screen, and placed in a climate-controlled dry cabinet for subsequent analysis.

### 2.2 Chemicals and equipments

Standards and NKA-II resin were purchased from Yuanye Bio-Technology Co., Ltd. (Shanghai, China). Ten ILs (listed in Table 1) were purchased from Aladdin (Shanghai, China). 2,2-Diphenyl-1-picrylhydrazyl (DPPH), Folin–Ciocalteu reagent, and 2,2′-azino-bis(3-ethylbenzothiazoline-6-sulfonic acid) (ABTS) were obtained from Sigma (St. Louis, MO, USA). Dulbecco’s modified Eagle medium (DMEM) and fetal bovine serum (FBS) were purchased from HyClone (Logan, UT, USA).

**Table 1:** Structural parameters of ionic liquids tested.

Ionic Liquids	Synonym	Molecular formula	Cation	Anion
1-Butyl-3-methylimidazolium dihydrogenphosphate	[BMIM]H_2_PO_4_	C_8_H_17_N_2_O_4_P		
1-Butyl-3-methylimidazolium trifluoromethanesulfonate	[BMIM]OTf	C_9_H_15_F_3_N_2_O_3_S		
1-Butyl-3-methylimidazolium tetrachloroferrate	[BMIM]FeCl_4_	C_8_H_15_Cl_4_FeN_2_		
1-Butyl-3-methylimidazolium tetrafluoroborate	[BMIM]BF_4_	C_8_H_15_BF_4_N_2_		
1-Butyl-3-methylimidazolium hexafluorophosphate	[BMIM]PF_6_	C_8_H_15_F_6_N_2_P		
1-Butyl-3-methylimidazolium chloride	[BMIM]Cl	C_8_H_15_ClN_2_		**·**Cl^–^
1-Butyl-3-methylimidazolium bromide	[BMIM]Br	C_8_H_15_BrN_2_		**·**Br^–^
1-Ethyl-3-methylimidazolium bromide	[EMIM]Br	C_6_H_11_BrN_2_		**·**Br^–^
1-Hexyl-3-methylimidazolium bromide	[HMIM]Br	C_10_H_19_BrN_2_		**·**Br^–^
1-Methyl-3-octylimidazolium bromide	[OMIM]Br	C_12_H_23_BrN_2_		**·**Br^–^

The extraction process was conducted using a KQ-500DB ultrasonic generator (Kunshan Ultrasonic Instruments Co. Ltd., Suzhou, China). Sample absorbance was measured using a SpectraMax® iD5 microplate reader (Molecular Devices, San Jose, CA, USA). Phenolic compounds were separated using a ZORBAX Eclipse Plus C18 column (1.8 μm, 2.1 × 100 mm) and quantified using a G6470B QqQ LC–MS system equipped with an ESI source (Agilent, Santa Clara, CA, USA).

### 2.3 Optimization of IL-UAE method

#### 2.3.1 Screening of ILs

A 1.0 mol/L solution of each IL was prepared, and 2.0 g of powdered *C. speciosa* leaves was added to each solution (20 mL). Three cycles of extraction were performed at an ultrasonic power of 300 W, each lasting 10 min. After extraction, the extracts from each cycle were combined and used for subsequent analysis.

#### 2.3.2 Single-factor design

Building on the results of IL screening, a single-factor design was used to determine the optimal range of four key factors affecting the CSL-TP yield: [BMIM]Br concentration, ultrasonic power, extraction time, and liquid-to-solid ratio. To evaluate the effect of [BMIM]Br concentration, IL-UAE was performed using [BMIM]Br concentrations ranging from 0 to 1.5 mol/L at 300 W for 10 min, with a liquid-to-solid ratio of 15 mL/g. The effect of ultrasonic power on the CSL-TP yield was assessed in the range of 200 to 500 W, with a constant [BMIM]Br concentration of 1.0 mol/L, a liquid-to-solid ratio of 15 mL/g, and an extraction time of 10 min. To evaluate the effect of extraction time, IL-UAE was performed for durations ranging from 5 to 30 min at 300 W, with a [BMIM]Br concentration of 1.0 mol/L and a liquid-to-solid ratio of 15 mL/g. Finally, the effect of the liquid-to-solid ratio was examined in the range of 10–30 mL/g at 300 W for 10 min, with the [BMIM]Br concentration fixed at 1.0 mol/L.

#### 2.3.3 Experimental design and analysis of the response surface

The insights gained from the single-factor tests provide a foundation for applying response surface methodology (RSM). At this stage, the range of four independent variables was further refined: *X*_1_ ([BMIM]Br concentration), *X*_2_ (ultrasonic power), *X*_3_ (extraction time), and *X*_4_ (liquid-to-solid ratio). A four-factor, three-level Box–Behnken design (BBD) was adopted to optimize these variables for maximising the CSL-TP yield (*Y*). Table S1 showed the settings and results of 29 randomised experiments conducted under the BBD framework. The data were analysed using the following polynomial model:

(1) $$Y=\alpha_{\text{0}}+\sum_{i=1}^{k}\alpha_{i}X_{i}+\sum_{i=1}^{k}\alpha_{\mathrm{ii}}X_{i}^{\text{2}}+\sum_{i=1}^{k}\sum_{j=i+1}^{k-1}\alpha_{\mathrm{ij}}X_{i}X_{j}$$

where *X_i_* and *X_j_* indicated the independent variables; *α*_0_ denoted the intercept and *α_i_*, *α_ii_* and *α_ij_* denoted the linear, quadratic and interaction coefficients, respectively. *k* denoted the number of variables.

An analysis of variance (ANOVA) test was used to evaluate the model’s performance. Key metrics included model *p*-value, lack-of-fit *p*-value, correlation coefficient (*R*^2^), adjusted *R*^2^, and coefficient of variance (C.V.). These metrics confirmed the adequacy of the model when representing the data. The optimal extraction parameters were determined using a numerical optimization method based on the desirability function criteria.

### 2.4 Other extraction methods

#### 2.4.1 Heat reflux extraction (HRE)

HRE was performed based on preliminary optimization results. Specifically, 2.0 g of powdered *C. speciosa* leaves were placed in a heat reflux extractor and extracted three times using 45 mL of 70 % ethanol for 2 h per cycle. The resulting extracts were combined and centrifuged at 13,000 rpm for 10 min before further analysis.

#### 2.4.2 UAE

The UAE was conducted based on preliminary optimization results. Briefly, 2.0 g of powdered *C. speciosa* leaves were placed in an ultrasonic generator (380 W, 40 kHz) and extracted three times using 45 mL of 70 % ethanol for 10 min per cycle. The resulting extracts were combined and centrifuged at 13,000 rpm for 10 min before further analysis.

### 2.5 Measurement of total phenolic content (TPC)

TPC was measured using the Folin–Ciocalteu method, as described in our previous study [3]. A 50 μL aliquot of the sample was mixed with 200 μL of tenfold-diluted Folin–Ciocalteu reagent. The mixture was allowed to react for 3 min before the addition of 1 mL of 7.5 % Na_2_CO_3_ solution. The solution was then mixed and left in the dark at room temperature for 60 min. Subsequently, a 250 μL aliquot of the reaction mixture was transferred to a 96-well plate, and its absorbance was measured at 760 nm. A calibration curve for gallic acid was constructed, yielding the linear equation *y* = 0.0022*x* + 0.0613 (*R*^2^ = 0.9997), where *y* represented the absorbance and *x* denoted the concentration in the range of 25 to 250 μg/mL.

### 2.6 Scanning electron microscopy (SEM) analysis

After extraction, *C. speciosa* leaves were thoroughly rinsed with ultrapure water and freeze-dried. The dried leaves were then coated with gold. The structural morphology of the leaves was examined using SEM at an acceleration voltage of 10 kV and image magnification of 100,000 × . High-resolution images were captured from randomly selected areas of each sample, ensuring that the dimensions chosen were representative of the entire sample.

### 2.7 Purification of CSL-TP by NKA-II resin column chromatography

Macroporous adsorbent resin was used to purify CSL-TP and facilitate the recovery of [BMIM]Br. NKA-II resin was packed into an open glass column (*φ* = 20 mm, diameter-to-height ratio = 1:6), resulting in an approximate bed volume (BV) of 38 mL. During adsorption, the crude extract was introduced into the column at flow rates of 1, 2, and 3 BV/h, and the concentration of CSL-TP in the column effluent was monitored. For desorption, the loaded column underwent gradient elution starting with H_2_O, followed by 10 %, 30 %, 50 %, 70 %, and 95 % ethanol solutions. Each gradient stage was applied for 6 BV at a flow rate of 2 BV/h, and the CSL-TP concentration in the column effluent was measured.

### 2.8 Quantitative analysis using UPLC–QqQ–MS/MS

#### 2.8.1 Analysis conditions

The injection volume was 1 μL, and the column temperature was maintained at 40 °C. The mobile phase comprised 0.1 % formic acid in water (phase A) and 0.1 % formic acid in acetonitrile (phase B), delivered at a flow rate of 0.4 mL/min. The gradient elution profile was as follows: from 0 to 6 min, phase B increased from 10 % to 25 %; from 6 to 8 min, phase B increased to 100 %; from 8 to 10 min, phase B remained constant at 100 %; from 10 to 11 min, phase B decreased to 10 % and from 11 to 12 min, phase B stabilised at 10 %. The conditions for the ESI source were as follows: gas temperature: 350 °C; gas flow rate: 10 L/min; sheath gas temperature: 350 °C; sheath gas flow rate: 8 L/min; nebuliser pressure: 45 psi; capillary voltage: 3500 V; and nozzle voltage: 1000 V. The optimized MRM parameters in negative ionisation mode were listed in Table S2.

#### 2.8.2 Method validation

In the validation process, linearity, linear range, limit of quantification (LOQ), limit of detection (LOD), precision, and recovery were evaluated. The LOD and LOQ were determined at signal-to-noise ratios of 3:1 and 10:1, respectively. The precision was determined by calculating the relative standard deviation (RSD) of the peak area signals for both intra-day and inter-day measurements. The intra-day precision was evaluated by analysing samples five times within a single day, whereas the inter-day precision was evaluated over five consecutive days. Recovery tests were performed using extracts spiked with reference standards.

### 2.9 Antioxidant activity

#### 2.9.1 DPPH• scavenging

The ability of CSL-TP to scavenge DPPH• was evaluated using the method described in a previous study [25]. Briefly, 1.0 mL of 0.1 mM DPPH solution was mixed with 3.0 mL of CSL-TP solution at concentrations ranging from 25 to 400 µg/mL in ethanol. The mixture was then shaken and incubated at room temperature for 30 min. After incubation, absorbance was measured at 517 nm, with ascorbic acid as the positive control. The DPPH• scavenging rate was calculated using Eq. (2):

(2) $$\text{Free radical scavenging rate (\textbackslash{}\%)}=\left(A_{0}-A_{s}\right)/A_{0}\times 100\%$$

where *A*_0_ and *A_s_* are the absorbances of the blank (without the phenolic extract) and sample solutions, respectively.

#### 2.9.2 ABTS^+^• scavenging

The ability of CSL-TP to scavenge ABTS^+^• was evaluated using the method outlined in a previous study [26]. A 7 mM ABTS solution in PBS at pH 7.4 was combined in equal volumes with a 2.45 mM K_2_S_2_O_8_ solution in the dark to generate ABTS^+^•. After 16 h of incubation, the absorbance at 734 nm was adjusted to 0.70 ± 0.02. Subsequently, 1.0 mL of the sample solution at concentrations ranging from 25 to 400 µg/mL was added to 4.0 mL of the ABTS^+^• solution. The mixture was incubated in the dark for 10 min, and absorbance was then measured at 734 nm. Trolox was used as the positive control, and the scavenging rate of ABTS^+^• radical was calculated using Eq. (2).

### 2.10 Anti-colorectal cancer activity

The anti-colorectal cancer activity of CSL-TP was evaluated using a previously described method [27]. HCT-116 and HT-29 cells were seeded in 96-well plates at a density of 1 × 10^4^ cells per well, with each well containing 100 µL of complete DMEM. The cells were incubated for 12 h at 37 °C in a humidified atmosphere containing 5 % CO_2_. After incubation, the medium was replaced with 100 µL of CSL-TP at concentrations ranging from 10 to 160 μg/mL. After 48 h of treatment, 10 μL of MTT solution (5 mg/mL) was added to each well, and the plates were incubated for an additional 4 h. The supernatant was then removed, and 150 µL of DMSO was added. The absorbance was measured at 570 nm. An untreated solution was used as the control under identical conditions. The percentage inhibition of cell proliferation was calculated using Eq. (3).

(3) $$\text{Inhibition (\textbackslash{}\%) =}\;\left(A_{c}-A_{s}\right)/A_{c}\times 100\%$$

where *A_c_* was the absorbance of the group untreated with CSL-TP, and *A_s_* was the absorbance of the group treated with CSL-TP.

### 2.11 Statistical analysis

The data were expressed as the mean ± SD and were derived from a minimum of three independent replicates. The experimental design of the response surface and data analysis were performed using the Design-Expert 13.0 software. The half-maximal inhibitory concentration (IC_50_) and significance difference analysis (LSD test, *p* < 0.05) were determined using IBM SPSS 27.0.1.0 software.

## 3 Results and discussion

### 3.1 Influence of ILs on CSL-TP yield

**Fig. 1:**
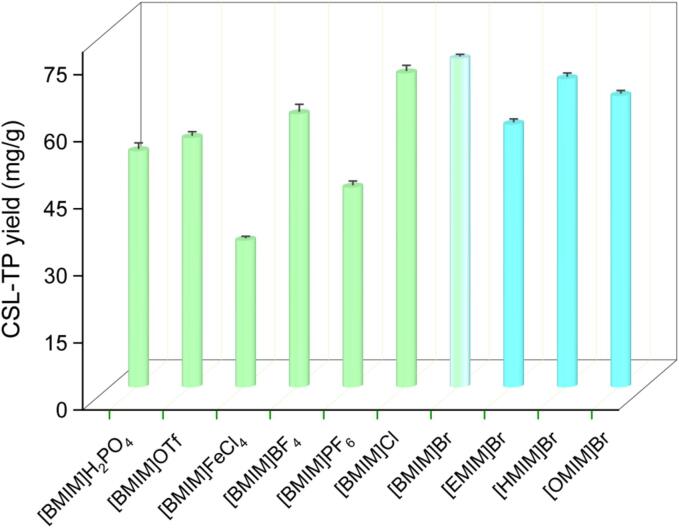
Effects of different ionic liquids on the CSL-TP yield.

The structure of ILs plays a crucial role in determining their physicochemical properties, which affect the extraction efficiency of target compounds. To identify the most suitable IL for the extraction of CSL-TP, ten 1-alkyl-3-methylimidazolium ILs with different anions and cations were evaluated. The results were shown in Fig. 1. The yield of CSL-TP extracted using ILs containing the same cation ([BMIM]^+^) but different anions, such as Br^−^, Cl^−^ and BF_4_^−^, was higher. This outcome could be attributed to enhanced interactions, such as π–π, ionic/charge–charge, and hydrogen bonding between [BMIM]Br, [BMIM]Cl, and [BMIM]BF_4_ and the phenolic compounds [28]. In contrast, the yield of CSL-TP extracted using ILs such as [BMIM]FeCl_4_, [BMIM]PF_6_, and [BMIM]H_2_PO_4_ was lower, which could be attributed to significant steric hindrance caused by the larger structures of their anionic functional groups [29]. The extraction efficiency of ILs with varying alkyl chain lengths but identical anion (Br^−^) was analysed. The yield of CSL-TP significantly increased from ethyl ([EMIM]Br) to butyl ([BMIM]Br). However, when the chain length was extended to hexyl ([HMIM]Br) and octyl ([OMIM]Br), the CSL-TP yield decreased. A similar trend has been reported for the IL-UAE of flavonoids from bamboo leaves [7]. In general, in imidazolium-based ILs, longer alkyl chains tend to reduce polarity by increasing hydrophobicity and shielding the polar properties of imidazolium rings. Based on the principle of compatibility, the polarity of [BMIM]Br is the most suitable for extracting phenolic compounds from *C. speciosa* leaves [15], [28]. Consequently, [BMIM]Br was selected as the solvent to extract CSL-TP for further investigation.

### 3.2 Analysis of single-factor experiment

#### 3.2.1 [bmim]br concentration

**Fig. 2:**
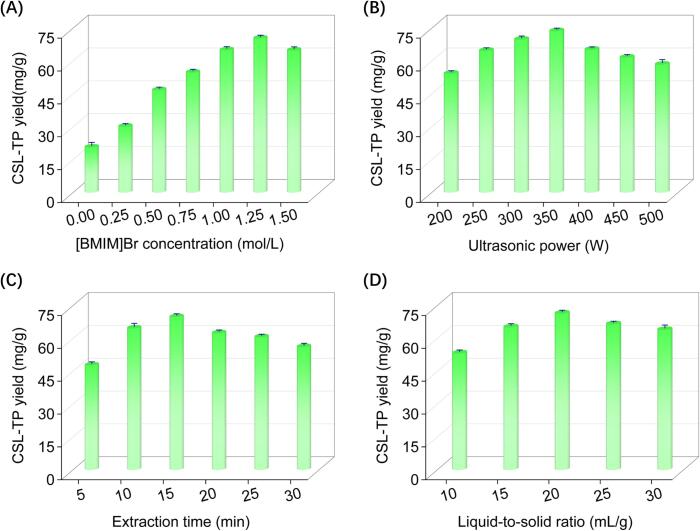
Effects of four independent variables on the CSL-TP yield. [BMIM]Br concentration (A), ultrasonic power (B), extraction time (C), and liquid-to-solid ratio (D).

To maximize the yield of CSL-TP, aqueous [BMIM]Br solutions with concentrations ranging from 0 to 1.5 mol/L were evaluated. As shown in Fig. 2**A**, increasing the concentration of [BMIM]Br up to 1.25 mol/L improved the CSL-TP yield. However, further increases resulted in diminished extraction performance. Similar trends have been reported for the IL-UAE of curcuminoid from *Curcuma longa* L. [17] and flavonoids from bamboo leaves [7]. Higher [BMIM]Br concentrations resulted in significant disruption of the cellulose matrix and enhanced the solubilisation of phenolics, thereby increasing the extraction efficiency. However, [BMIM]Br concentrations above 1.25 mol/L increased its viscosity, which hindered the mass transfer of the phenolic compounds, weakened cavitation caused by ultrasonic vibrations and reduced the efficiency of extraction [17]. Consequently, a 1.25 mol/L aqueous [BMIM]Br solution was identified as the most suitable extractant.

#### 3.2.2 Ultrasonic power

The effect of ultrasonic power on the CSL-TP yield was investigated in the range of 200–500 W. Optimizing the ultrasonic power is crucial for achieving satisfactory yields when using the IL-UAE method. As shown in Fig. 2**B**, the CSL-TP yield increased with increasing ultrasonic power, reaching a maximum at 350 W and then decreasing. A similar trend has been reported for the IL-UAE of saponins from *Platycodon grandiflorum* [15]. Increasing the ultrasonic power enhances the extraction efficiency and yield by improving the solvent–sample contact via cavitation effects. However, excessive ultrasonic power can degrade target compounds, reducing extraction yields and compromising the quality of the extracted product [30]. Based on these findings, the optimal ultrasonic power was determined as 350 W.

#### 3.2.3 Extraction time

The effect of extraction time, which is a crucial process parameter, on CSL-TP yield was evaluated from 5 to 30 min. As shown in Fig. 2**C**, the CSL-TP yield increased with prolonged extraction from 5 to 15 min. However, the yield decreased when the extraction duration exceeded 15 min. Similar trends have been reported for the IL-UAE of flavonoids from bamboo leaves [7] and saponins from *Platycodon grandiflorum* [15]. The initial increase in CSL-TP yield could be attributed to enhanced interactions between aqueous [BMIM]Br solution and phenolic compounds, facilitating the release of phenolic compounds from the plant matrix. However, extending the extraction duration beyond 15 min led to the increased dissolution of impurities and degradation of target compounds, resulting in reduced CSL-TP yield [31]. Consequently, the optimal extraction time was determined as 15 min.

#### 3.2.4 Liquid-to-solid ratio

The effect of the liquid-to-solid ratio on the CSL-TP yield was evaluated in the range of 10–30 mL/g. As shown in Fig. 2**D**, CSL-TP yield increased with a liquid-to-solid ratio between 10 and 20 mL/g. However, it decreased when the ratio exceeded 20 mL/g. A similar observation has been reported for the IL-UAE of flavonoids from bamboo leaves [7]. In general, a higher liquid-to-solid ratio enhances the dissolution of target compounds by increasing the concentration gradient and contact area between the solvent and solid, thereby improving the extraction process and yield. However, excessively high liquid-to-solid ratios may result in the co-extraction of impurities, negatively affecting the purity and quality of the target compounds. In addition, a higher liquid-to-solid ratio reduces the ultrasonic energy density in the extractant, which can affect yield [32]. Based on these findings, a liquid-to-solid ratio of 20 mL/g was determined to be optimal.

### 3.3 RSM modelling and optimization of IL-UAE conditions

#### 3.3.1 Model fitting

The experimental data collected under various conditions in the BBD are summarized in Table S1. The CSL-TP yield ranged from 61.17 to 77.13 mg/g. Multiple regression analysis was then performed to provide valuable insights for optimizing the IL-UAE parameters and enable the prediction of complex relationships between the independent variables and the dependent variable. For the resulting model, a quadratic polynomial equation was used, as shown in Eq. (4).

(4) $$\begin{array}{c} Y=76.46-1.24X_{1}+1.31X_{2}-1.59X_{3}-1.29X_{4}+1.69X_{1}X_{2}-3.01X_{1}X_{3}+0.64X_{1}X_{4}\\ -3.44X_{2}X_{3}+2.84X_{2}X_{4}-4.00X_{3}X_{4}-4.62X_{1}^{2}-5.53X_{2}^{2}-1.89X_{3}^{2}-4.68X_{4}^{2} \end{array}$$

In addition, ANOVA was conducted to evaluate the variance and adequacy of the quadratic model. As presented in Table S3, the model was statistically significant (*p* < 0.0001), whereas the ‘lack of fit’ was not statistically significant (*p* > 0.05), confirming the suitability and reliability of the prediction model [33]. All linear, quadratic, and interaction coefficients, except for the *X*_1_*X*_4_ term, were significant (*p* < 0.05). The *R*^2^ value was 0.9582, indicating that the regression model explained 95.82 % of the total variance in the data. The adjusted *R*^2^ value of 0.9164, further supported the robustness of this model, demonstrating a significant relationship between the experimental and predicted outcomes. Additionally, the C.V.% was 2.02, indicating that the model exhibited satisfactory repeatability and consistency across multiple trials [34].

#### 3.3.2 Analysis of the response surface

**Fig. 3:**
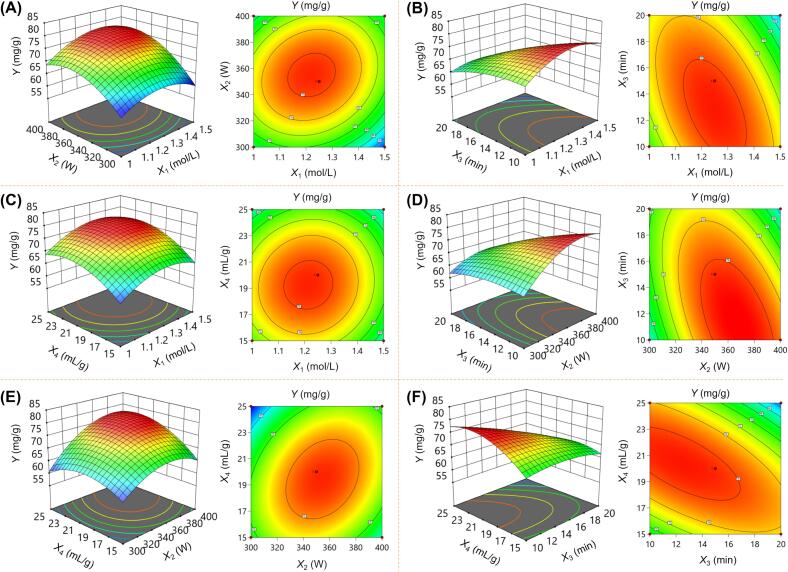
Three-dimensional response surface plots and the two-dimensional contour plots illustrating the interactions between *X*_1_ and *X*_2_ (A), *X*_1_ and *X*_3_ (B), *X*_1_ and *X*_4_ (C), *X*_2_ and *X*_3_ (D), *X*_2_ and *X*_4_ (E), and *X*_3_ and *X*_4_ (F) on the CSL-TP yield.

The response surfaces provide an intuitive visual representation of the interaction between two factors, particularly when the other factors are held constant at zero. Steeper surfaces and elliptical contour plots indicate a more pronounced interaction between the independent variables and the dependent variable, whereas gently sloping surfaces and circular contours indicate a weaker interaction between the two factors [35]. Fig. 3 showed that the CSL-TP yield initially increased and then decreased with the increase in the three independent variables, *X*_1_, *X*_2_ and *X*_4_, which was consistent with the results of the single-factor tests. However, the highest CSL-TP yield was obtained only when *X*_3_ was set to a minimum value of 10 min. Based on the three-dimensional response surface and two-dimensional contour plots, the interaction between *X*_3_ and *X*_4_ (Fig. 3**F**) exhibited the strongest effect on the CSL-TP yield, followed by the interactions between *X*_2_ and *X*_3_ (Fig. 3**D**) and between *X*_1_ and *X*_3_ (Fig. 3**B**). In contrast, the interaction between *X*_1_ and *X*_4_ was the weakest (Fig. 3**C**), indicating that these factors have a minimal effect on the CSL-TP yield when considered together. These findings are consistent with the analysis results presented in Table S3, further validating the observed interactions.

#### 3.3.3 Process optimization and validation

Based on the desirability function derived from the BBD, the optimal combination of independent variables for achieving a predicted maximum CSL-TP yield of 78.58 mg/g was as follows: a [BMIM]Br concentration of 1.33 mol/L, ultrasonic power of 380.43 W, extraction time of 10 min, and liquid-to-solid ratio of 22.49 mL/g. To improve the practicality of the operation, the IL-UAE of CSL-TP was performed under slightly modified conditions: a [BMIM]Br concentration of 1.33 mol/L, an ultrasonic power of 380 W, an extraction time of 10 min and a liquid-to-solid ratio of 22 mL/g. These optimized conditions resulted in a CSL-TP yield of 78.14 ± 0.35 mg/g, which closely aligned with the predicted value, confirming that the developed predictive model was reliable and stable.

### 3.4 Comparative analysis of different extraction methods

**Fig. 4:**
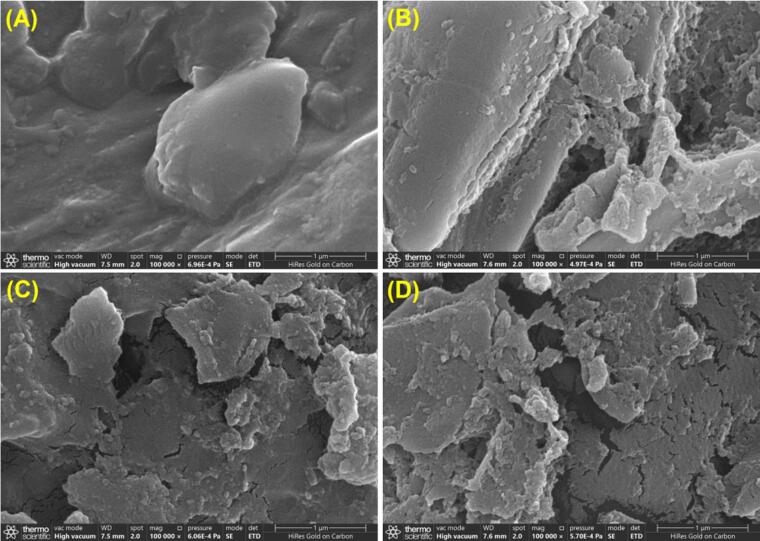
SEM images of *C. speciosa* leaves before (A) and after treatment with HRE (B), UAE (C), and IL-UAE (D).

HRE using 70 % ethanol for 2 h and UAE using 70 % ethanol for 10 min resulted in CSL-TP yields of 51.27 ± 0.78 and 64.50 ± 0.49 mg/g, respectively, with the same liquid-to-solid ratio and extraction cycles as IL-UAE. Both yields were significantly lower than those obtained via IL-UAE. Similar trends have been reported for the extraction of biphenyl cyclooctene lignans from *Schisandra chinensis* Baill. fruits [28] and curcuminoids from *Curcuma longa* L. [17]. SEM was used to observe morphological changes in *C. speciosa* leaves before and after treatment (Fig. 4). The surface of the untreated leaves was relatively smooth with no debris or pores. However, after extraction, the surface structure of *C. speciosa* leaves was compromised, showing varying degrees of damage to the cell walls and resulting in the formation of debris and cavities. The most significant damage was observed in the leaves from which TP was extracted using IL-UAE, followed by those from which TP was extracted using UAE.

Differences in extraction yields could be attributed to the inherent properties of solvents, extraction methods, and physicochemical properties of the target compounds. These findings highlight the advantages of using [BMIM]Br over an aqueous ethanol solution for extraction, as well as the superior performance of UAE compared to conventional RAE. During UAE, high-frequency sound waves were used to generate collapsing cavitation bubbles, which create localised heat and pressure that disrupt plant tissue and enhance the diffusion of phenolic compounds, improving extraction efficiency [36]. In contrast, an aqueous [BMIM]Br solution exhibited greater affinity for the active sites of plant cellulose than aqueous ethanol solution, potentially disrupting the hydrogen bond network and enhancing the dissolution of solutes within the cells. Furthermore, the nitrogen atoms in imidazolium-based [BMIM]Br can form hydrogen bonds with hydroxyl radicals in phenolic compounds, enhancing solvent selectivity and improving extraction rates [17]. Therefore, UAE using [BMIM]Br represents a green and effective approach for extracting phenolic compounds from *C. speciosa* leaves.

### 3.5 Purification of CSL-TP by NKA-II resin column chromatography

#### 3.5.1 Leakage curves

**Fig. 5:**
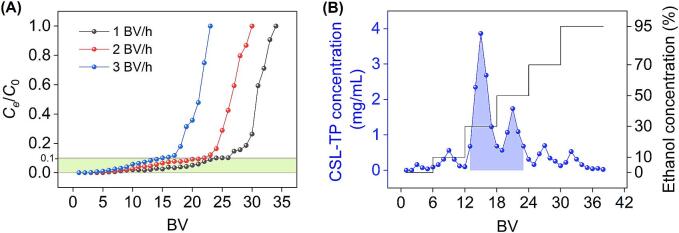
Leakage curves (A) and elution curves (B) for CSL-TP on the NKA-II resin column.

Leakage curves in column chromatography are essential for optimizing feed rates and sample volumes to enhance purification efficiency. As shown in Fig. 5**A**, leakage points, defined as the CSL-TP concentration in the effluent that reached 10 % of the concentration in the sample solution, emerged rapidly at higher feed rates. A similar trend was observed in our study on the purification of phenolic compounds from *Salvia miltiorrhiza* Bunge leaves [32]. This could be attributed to the longer interaction time between the phenolic compounds and the resin at lower feed rates, which enhanced the adsorption [3]. At the leakage point, the feed volumes at flow rates of 1, 2, and 3 BV/h were 24, 22, and 15 BV, respectively. Although the feed volume at 1 BV/h was slightly higher than that at 2 BV/h, the operational efficiency indicated that a feed rate of 2 BV/h, with a corresponding volume of 22 BV, was most suitable.

#### 3.5.2 Elution curves

The analysis of elution curves facilitates the determination of the optimal elution flow rate and solvent gradient, ensuring the efficient purification of target compounds while providing critical retention times for the precise collection of fractions. The desorption curve at a flow rate of 2 BV/h, as shown in Fig. 5**B**, revealed that 6 BV of H_2_O and 6 BV of 10 % ethanol were insufficient to desorb the phenolic compounds. Therefore, these solvents were primarily used to remove polar impurities. Subsequently, 6 BV of 30 % ethanol yielded a fraction rich in CSL-TP, whereas 6 BV of 50 % ethanol eluted an additional amount of phenolic compounds. At 70 % and 95 % ethanol concentrations, only trace amounts of the remaining phenolic compounds were eluted. Based on these results, the eluate was collected between 13 and 23 BV, as indicated by the shaded region in Fig. 4**B**. This fraction was then concentrated under vacuum and freeze-dried, yielding a phenolic extract with a purity of 75.40 % ± 1.93 %. The recovery rate of CSL-TP was 84.51 %, whereas that of [BMIM]Br was 96.32 %.

### 3.6 Quantitative analysis of major phenolic compounds

**Fig. 6:**
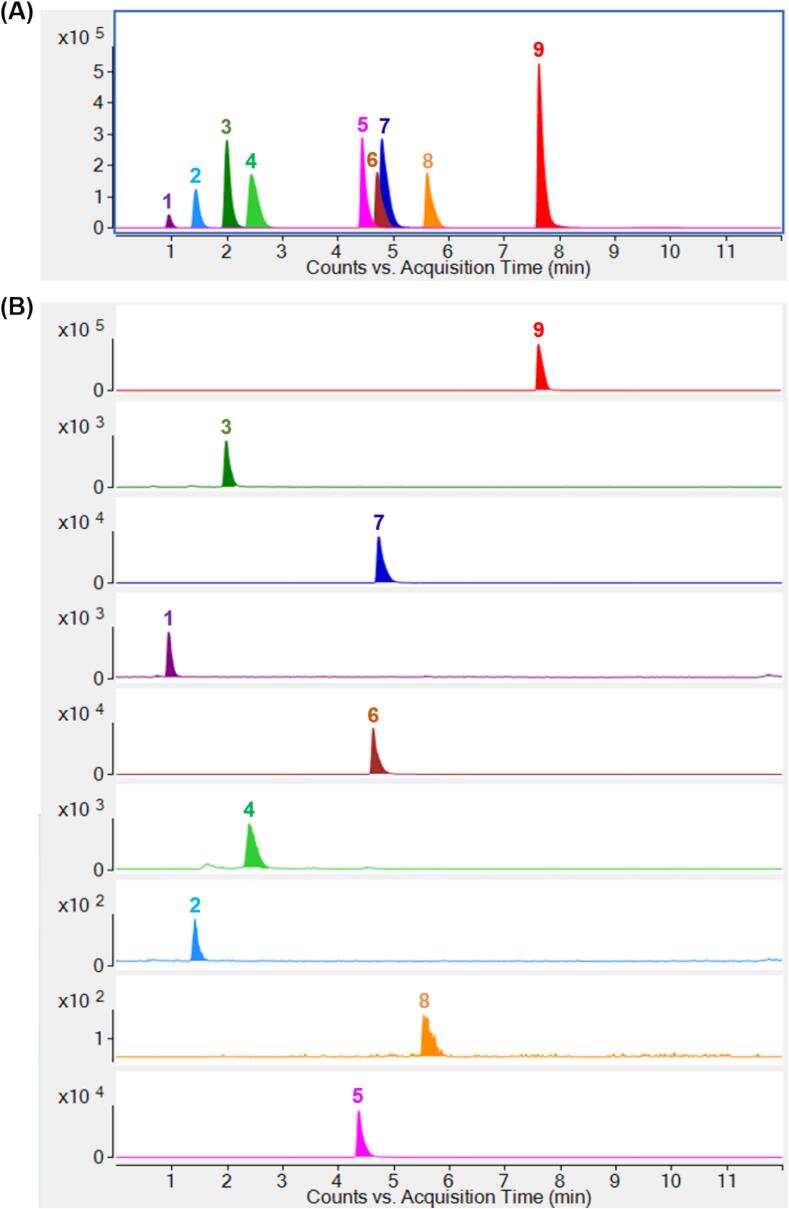
Chromatogram of extracted MRM transitions from a mixture of nine phenolic standards (A) and representative chromatograms of extracted MRM transitions from CSL-TP extracts showing nine phenolic compounds (B). Peaks: (1) gallic acid, (2) protocatechuic acid, (3) chlorogenic acid, (4) proanthocyanidin B2, (5) rutin, (6) isoquercetin, (7) cynaroside, (8) quercitrin, and (9) apigenin.

As outlined in Section 2.8.1, the conditions of UPLC–QqQ–MS/MS for the simultaneous quantification of nine major phenolic compounds including gallic acid, protocatechuic acid, chlorogenic acid, proanthocyanidin B2, rutin, isoquercitin, cynaroside, quercitrin, and apigenin, were systematically optimized. UPLC parameters such as injection volume, column temperature, mobile phase composition and elution procedure were fine-tuned to enhance chromatographic separation. In addition, the MRM parameters, including the collision energy and fragmentor settings, were adjusted to maximize the analytical sensitivity, with the most responsive transition selected for quantification. Fig. 6**A and B** show the extracted MRM chromatograms of a standard phenolic mixture and CSL-TP extracts, respectively. As shown in Table 2, all target compounds exhibited excellent linearity (*R*^2^ ≥ 0.998) across the tested concentration range. The LODs ranged from 2.52 to 10.01 ng/mL, and the LOQs ranged from 7.42 to 32.85 ng/mL. The intraday and interday RSDs were ≤ 2.63 % and ≤ 4.12 %, respectively, with recoveries ranging between 97.15 % ± 0.83 % and 103.28 % ± 0.91 %. The developed UPLC–QqQ–MS/MS method was suitable for the simultaneous quantification of nine phenolic compounds, making it invaluable for controlling the quality of purified CSL-TP extracts. Notably, chlorogenic acid (12.32 % ± 0.76 %) was the most abundant compound, followed by cynaroside (8.57 % ± 0.39 %) and rutin (8.35 % ± 0.60 %). The nine phenolic compounds accounted for approximately 50.63 % of the total phenolic content.

**Table 2:** Method validation for the UPLC-QqQ-MS/MS quantification of nine phenolics in the purified CSL-TP extracts.

Phenolic compounds	Regression equation	Linear range (ng/mL)	*R*^2^	LOD (ng/mL)	LOQ (ng/mL)	Precision (RSD, %)		Recovery rate (%)	Content (%)
						Intra-day	Inter-day		
Gallic acid	*y* = 106.80*x*-63.14	50–500	0.9998	2.52	7.42	0.91	1.53	99.47 ± 1.85	3.05 ± 0.24
Protocatechuic acid	*y* = 29.62*x* + 77.90	50–500	0.9997	10.01	32.85	1.40	3.28	102.53 ± 1.72	4.63 ± 0.41
Chlorogenic acid	*y* = 53.98*x* + 1632.55	40–2000	0.9989	6.20	16.20	0.75	2.24	103.04 ± 0.94	12.32 ± 0.76
Proanthocyanidin B2	*y* = 31.24*x* + 95.79	50–1000	0.9995	9.76	28.55	2.46	4.02	98.96 ± 0.77	4.74 ± 0.29
Rutin	*y* = 80.31*x*-124.62	50–1000	0.9994	5.85	16.30	2.25	3.97	97.15 ± 0.83	8.35 ± 0.60
Isoquercetin	*y* = 46.74*x* + 116.13	25–500	0.9997	4.92	15.72	1.61	4.12	98.50 ± 0.52	2.60 ± 0.17
Cynaroside	*y* = 62.81*x* + 54.95	25–1000	0.9988	3.75	12.44	1.39	3.85	101.60 ± 1.27	8.57 ± 0.39
Quercitrin	*y =* 74.86*x*-105.32	25–500	0.9992	4.80	11.26	2.63	3.41	103.28 ± 0.91	0.93 ± 0.07
Apigenin	*y =* 51.63*x* + 82.47	40–2000	0.9990	5.15	17.12	1.90	3.56	98.69 ± 1.21	5.44 ± 0.35

*Notes*: LOD – limit of detection; LOQ – limit of quantification; RSD – relative standard deviation; *R*^2^ – correlation coefficients.

### 3.7 Bioactivity assessment

#### 3.7.1 Antioxidant activity

**Fig. 7:**
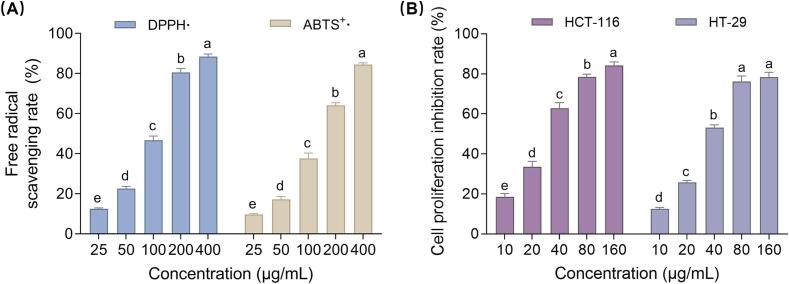
Antioxidant and anti-colorectal cancer activities of CSL-TP extracts. Scavenging capacity against DPPH• and ABTS^+^• (A); Inhibiting the proliferation of HCT-116 and HT-29 cells (B). Different letters indicate significant differences as determined by LSD test (*p* < 0.05).

The DPPH• and ABTS^+^• radical scavenging assays are widely used to evaluate the antioxidant activity of natural products. Phenolic compounds are known for their exceptional antioxidant properties. In this study, the antioxidant activity of CSL-TP extracts was assessed using these assays at concentrations ranging from 25 to 400 μg/mL. As shown in Fig. 7**A,** the purified CSL-TP extracts exhibited a concentration-dependent scavenging effect on DPPH• and ABTS^+^• radicals. The IC_50_ value of CSL-TP for DPPH• scavenging was 102.53 µg/mL, whereas that of ascorbic acid was 45.91 µg/mL. Similarly, the IC_50_ value of CSL-TP for ABTS^+^• scavenging was 134.77 µg/mL, whereas that of Trolox was 86.20 µg/mL. These results indicated that the purified CSL-TP extracts exhibited significant antioxidant activity and showed potential as natural antioxidants.

#### 3.7.2 Anti-colorectal cancer activity

Colorectal cancer is one of the most prevalent cancers worldwide, with its incidence steadily increasing [37]. Phenolic compounds have shown promise as potential interventions for colorectal cancer treatment [38]. This study evaluated the antiproliferative activity of purified CSL-TP extracts against HCT-116 and HT-29 cells. As shown in Fig. 7**B**, the CSL-TP extracts demonstrated a concentration-dependent inhibition of HCT-116 cell proliferation in the range of 10–160 μg/mL, achieving an inhibition rate of 84.31 % ± 1.64 % at 160 μg/mL and an IC_50_ value of 31.27 μg/mL. Similarly, for HT-29 cells, the CSL-TP extract inhibited proliferation at concentrations ranging from 10 to 80 μg/mL, reaching an inhibition rate of 78.43 % ± 2.38 % at 80 μg/mL, with an IC_50_ value of 41.82 μg/mL. These results indicated that the purified CSL-TP extracts had significant antiproliferative activity and could serve as a therapeutic alternative for colorectal cancer.

## 4 Conclusions

This study was the first to report the use of the IL-UAE method to extract CSL-TP. The optimized process parameters were: [BMIM]Br concentration of 1.33 mol/L, extraction time of 10 min, ultrasonic power of 380 W, and liquid-to-solid ratio of 22 mL/g. Under these conditions, a CSL-TP yield of 78.14 ± 0.35 mg/g was achieved, which is significantly higher than that obtained via conventional HRE and UAE methods. SEM analysis confirmed the effectiveness of the IL-UAE method. A column chromatography method was developed using NKA-II resin for the efficient purification of crude CSL-TP extracts. A UPLC–QqQ–MS/MS method was established for simultaneous quantification of nine phenolic compounds and to ensure the high quality of the purified CSL-TP extracts. Bioactivity evaluations demonstrated that the purified CSL-TP extracts exhibited notable antioxidant activity and effectively inhibited the growth of human colorectal cancer cell lines. These findings provided a strong foundation for advancing research and practical applications of *C. speciosa* leaves in the pharmaceutical and food industries.

## CRediT authorship contribution statement

**Mengyang Hou:** Writing – original draft, Methodology, Investigation, Data curation, Conceptualization. **Chengyuan Lin:** Project administration, Conceptualization. **Lin Zhu:** Writing – review & editing, Validation, Funding acquisition. **Zhaoxiang Bian:** Writing – review & editing, Resources, Project administration, Funding acquisition.

## Declaration of competing interest

The authors declare that they have no known competing financial interests or personal relationships that could have appeared to influence the work reported in this paper.
