# Supplementary material for: Phenolics from Chaenomeles speciosa leaves: Ionic liquid-based ultrasound-assisted extraction, adsorptive purification, UPLC–QqQ–MS/MS quantification, and bioactivity assessment
Source: Ultrason Sonochem. 2025 Feb 16;114:107282. doi: 10.1016/j.ultsonch.2025.107282 (PMC11891731; doi:10.1016/j.ultsonch.2025.107282)
Supplement: Supplementary Data 1 [file mmc1.docx]

Supplementary Material for

**Phenolics from *Chaenomeles speciosa* leaves: Ionic liquid-based ultrasound-assisted extraction, adsorptive purification, UPLC-QqQ-MS/MS quantification, and bioactivity assessment**

Mengyang Hou ^a^, Chengyuan Lin ^a^, Lin Zhu ^a,b,^*, Zhaoxiang Bian ^a,b,^*

*^a^ Centre for Chinese Herbal Medicine Drug Development, Hong Kong Baptist University, Hong Kong 999077, PR China*

*^b^ School of Chinese Medicine, Hong Kong Baptist University, Hong Kong 999077, PR China*

* Corresponding authors:

1. *mail addresses*: [zhulin@hkbu.edu.hk](mailto:zhulin@hkbu.edu.hk) (L. Zhu), [bzxiang@hkbu.edu.hk](mailto:bzxiang@hkbu.edu.hk) (Z. Bian)

**Captions for Tables:**

**Table S1** BBD and corresponding response values.

**Table S2** Optimized MRM parameters for quantification of phenolics.

**Table S3** ANOVA for quadratic model.

**Table S1**

BBD and corresponding response values.

| Run | *X*_1_ (mol/L) | *X*_2_ (W) | *X*_3_ (min) | *X*_4_ (mg/L) | *Y* (mg/g) |
| --- | --- | --- | --- | --- | --- |
| 1 | 1.25 | 300 | 15 | 25 | 61.17 |
| 2 | 1.5 | 350 | 15 | 15 | 66.82 |
| 3 | 1.0 | 400 | 15 | 20 | 67.42 |
| 4 | 1.5 | 350 | 20 | 20 | 64.32 |
| 5 | 1.5 | 300 | 15 | 20 | 62.64 |
| 6 | 1.0 | 350 | 20 | 20 | 72.24 |
| 7 | 1.25 | 400 | 10 | 20 | 76.14 |
| 8 | 1.0 | 350 | 15 | 15 | 71.75 |
| 9 | 1.0 | 300 | 15 | 20 | 67.92 |
| 10 | 1.25 | 350 | 15 | 20 | 76.05 |
| 11 | 1.5 | 350 | 10 | 20 | 73.55 |
| 12 | 1.25 | 350 | 15 | 20 | 77.01 |
| 13 | 1.25 | 350 | 20 | 25 | 65.38 |
| 14 | 1.25 | 400 | 15 | 15 | 65.50 |
| 15 | 1.25 | 350 | 15 | 20 | 77.05 |
| 16 | 1.5 | 350 | 15 | 25 | 63.18 |
| 17 | 1.25 | 400 | 15 | 25 | 68.01 |
| 18 | 1.25 | 300 | 15 | 15 | 70.02 |
| 19 | 1.25 | 300 | 20 | 20 | 68.17 |
| 20 | 1.25 | 400 | 20 | 20 | 65.11 |
| 21 | 1.25 | 350 | 15 | 20 | 77.13 |
| 22 | 1.25 | 350 | 10 | 15 | 67.21 |
| 23 | 1.0 | 350 | 10 | 20 | 69.42 |
| 24 | 1.25 | 300 | 10 | 20 | 65.43 |
| 25 | 1.25 | 350 | 15 | 20 | 75.04 |
| 26 | 1.25 | 350 | 10 | 25 | 75.56 |
| 27 | 1.5 | 400 | 15 | 20 | 68.89 |
| 28 | 1.25 | 350 | 20 | 15 | 73.02 |
| 29 | 1.0 | 350 | 15 | 25 | 65.56 |

*Notes*: *X*_1_ – [BMIM]Br concentration; *X*_2_ – ultrasonic power; *X*_3_ – extraction time; *X*_4_ – liquid-to-solid ratio; *Y* – total phenolics yield

**Table S2**

Optimized MRM parameters for quantification of phenolics.

| Compounds | Formula | Retention time (min) | Precursor Ion (m/z) | Product Ion (m/z) | Fragmentor (V) | Collision Energy (eV) |
| --- | --- | --- | --- | --- | --- | --- |
| Gallic acid | C_7_H_6_O_5_ | 0.944 | 169.01 | 125 | 124 | 13 |
| Protocatechuic acid | C_7_H_6_O_4_ | 1.431 | 153.02 | 109.1 | 112 | 13 |
| Chlorogenic acid | C_16_H_18_O_9_ | 1.993 | 353.09 | 191.0 | 102 | 21 |
| Proanthocyanidin B2 | C_30_H_26_O_12_ | 2.435 | 577.13 | 407.1 | 60 | 25 |
| Rutin | C_27_H_30_O_16_ | 4.435 | 609.14 | 300.0 | 238 | 41 |
| Isoquercetin | C_21_H_20_O_12_ | 4.704 | 463.09 | 300 | 228 | 29 |
| Cynaroside | C_21_H_20_O_11_ | 4.791 | 447.09 | 285.1 | 233 | 29 |
| Quercitrin | C_21_H_20_O_11_ | 5.604 | 447.09 | 300 | 228 | 29 |
| Apigenin | C_15_H_10_O_5_ | 7.623 | 269.04 | 117.1 | 166 | 37 |

**Table S3**

ANOVA for quadratic model.

| Source | Sum of Squares | df | Mean Square | *F*-value | *p*-value |
| --- | --- | --- | --- | --- | --- |
| Model | 631.24 | 14 | 45.09 | 22.92 | < 0.0001 |
| *X*_1_ | 18.53 | 1 | 18.53 | 9.42 | 0.0083 |
| *X*_2_ | 20.59 | 1 | 20.59 | 10.47 | 0.0060 |
| *X*_3_ | 30.31 | 1 | 30.31 | 15.40 | 0.0015 |
| *X*_4_ | 19.92 | 1 | 19.92 | 10.12 | 0.0067 |
| *X*_1_*X*_2_ | 11.39 | 1 | 11.39 | 5.79 | 0.0305 |
| *X*_1_*X*_3_ | 36.30 | 1 | 36.30 | 18.45 | 0.0007 |
| *X*_1_*X*_4_ | 1.63 | 1 | 1.63 | 0.8262 | 0.3788 |
| *X*_2_*X*_3_ | 47.40 | 1 | 47.40 | 24.09 | 0.0002 |
| *X*_2_*X*_4_ | 32.26 | 1 | 32.26 | 16.40 | 0.0012 |
| *X*_3_*X*_4_ | 63.92 | 1 | 63.92 | 32.49 | < 0.0001 |
| *X*_1_^2^ | 138.18 | 1 | 138.18 | 70.23 | < 0.0001 |
| *X*_2_^2^ | 198.13 | 1 | 198.13 | 100.70 | < 0.0001 |
| *X*_3_^2^ | 23.06 | 1 | 23.06 | 11.72 | 0.0041 |
| *X*_4_^2^ | 142.18 | 1 | 142.18 | 72.26 | < 0.0001 |
| Residual | 27.55 | 14 | 1.97 |  |  |
| Lack of fit | 24.26 | 10 | 2.43 | 2.96 | 0.1539 |
| Pure error | 3.28 | 4 | 0.8210 |  |  |
| Cor total | 658.78 | 28 |  |  |  |

*Notes*: *X*_1_ – [BMIM]Br concentration; *X*_2_ – ultrasonic power; *X*_3_ – extraction time; *X*_4_ – liquid-to-solid ratio; df – degrees of freedom
